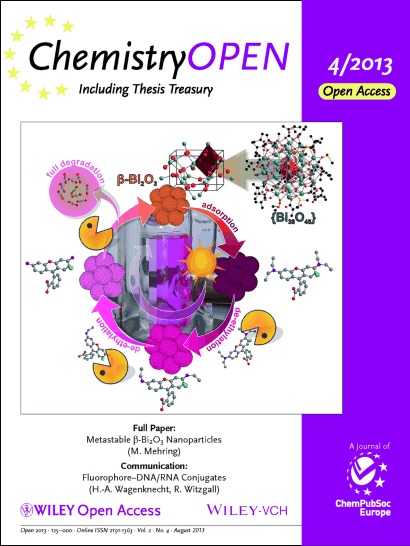# Metastable β-Bi_2_O_3_ Nanoparticles with Potential for Photocatalytic Water Purification Using Visible Light Irradiation

**DOI:** 10.1002/open.201300032

**Published:** 2013-08-21

## Abstract

Invited for this month′s cover is the group of Prof. Michael Mehring. The cover picture shows the degradation of the model dye rhodamine B (RhB) in water using β-Bi_2_O_3_ nanoparticles as photocatalyst, which was prepared from pre-organized bismuth oxido clusters. For more details, see the Full Paper on p. 146 ff.


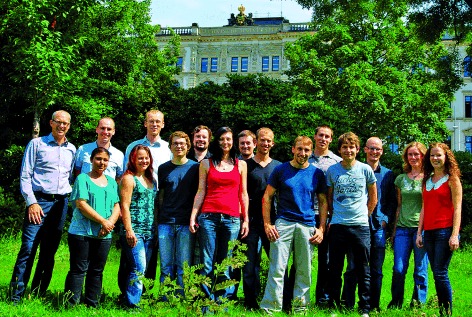


The Group of Michael Mehring


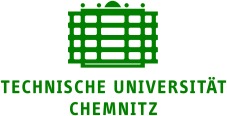


Technische Universität Chemnitz Fakultät für Naturwissenschaften, Institut für Chemie Professur Koordinationschemie 09111 Chemnitz (Germany) E-mail: michael.mehring@chemie.tu-chemnitz.de

## What aspects of this project do you find most exciting?

At least for us, nanoscaled bismuth oxido clusters are not only beautiful molecules but they also efficiently serve as single-source precursors for metastable β-Bi_2_O_3_ nanoparticles under mild synthetic conditions. The composition of the well-defined starting material as well as the work-up procedure strongly influence the photocatalytic decomposition process of the model dye rhodamine B (RhB), as shown in current and in very recently published studies (see *Dalton Trans.*­ **2013**, *42*, 1047–1056). However, it is demonstrated here that de-ethylation of RhB is always the initiating step, taking place on the bismuth oxide surface, but desorption of partially de-ethylated products is not necessarily observed. Our findings open opportunities to use clusters as starting materials and model compounds to better understand photocatalytic processes.

## What prompted you to investigate this topic/problem?

Water, the most important resource worldwide, is becoming more and more contaminated by chemical products, but established water purification processes are not exhaustively accessible. There is a strong need to develop cheap, efficient and decentralized water purification processes that make use of alternative energy sources. A potential approach is provided by sun-power-driven photocatalysis. However, additional research in this field is crucial to provide efficient, nontoxic and recyclable materials other than TiO_2_. Bismuth, though a heavy metal, is known for its low toxicity, making bismuth-based materials an interesting target for “green chemistry” approaches in materials science. Among others, the easily accessible nanoscaled β-Bi_2_O_3_, which is known for its activity under visible light irradiation, attracted our attention, not least due to its structural relationship with nanoscaled bismuth oxido clusters that we have been studying for some time now.

## What other topics are you working on at the moment?

We are interested in the development of strategies to synthesize new molecules and to transform such molecular precursors into more complex materials with potential applications such as catalysis, energy storage and energy conversion. Our current projects aim towards a more detailed understanding of the basic transformations on the molecular level. Most of the projects are based on main group metal chemistry (group 14 and 15), and four main areas of interest are: photovoltaic and photocatalytic materials, nanoscaled organic-inorganic hybrid materials via twin polymerization, novel organometallic and coordination compounds, and metal oxides and hybrid materials prepared from metal oxido clusters. Recently, we launched a project that is designated to water purification and water splitting, and first results are reported in this issue.